# Supplementary material for: Gene Editing of Nicotiana benthamiana Architecture for Space‐Efficient Production of Recombinant Proteins in Closed Environments
Source: Plant Biotechnol J. 2026 May 14;24(11):5600–15. doi: 10.1111/pbi.70685 (PMC13399606; doi:10.1111/pbi.70685)
Supplement: Supplementary file 1 [file PBI-24-5600-s001.pdf]

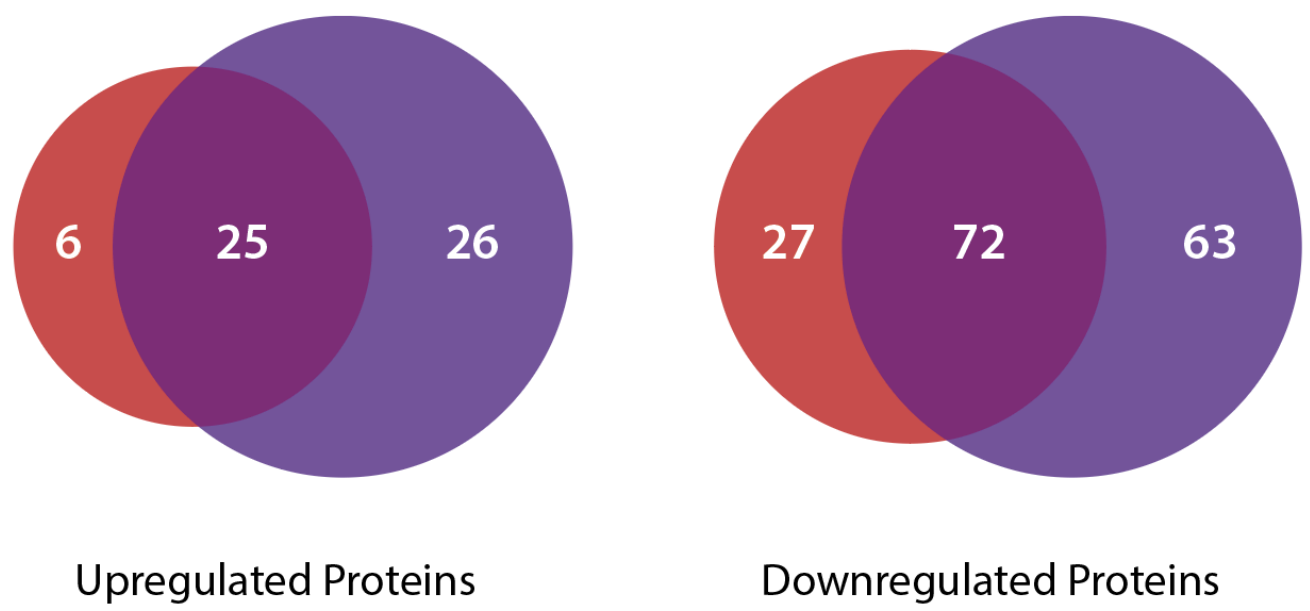

FIGURE S1 Venn diagram for the numbers of proteins up- or downregulated in Leaf P10 of mutant lines ΔCCD7–R and ΔCCD8. All proteins counted for this figure were up- or downregulated by at least 50% compared to the wild-type (WT), as inferred from limma p-values with an α threshod of 0.05. Specific details on the regulation trends of each protein are given in supplemental **Dataset S1**, online.

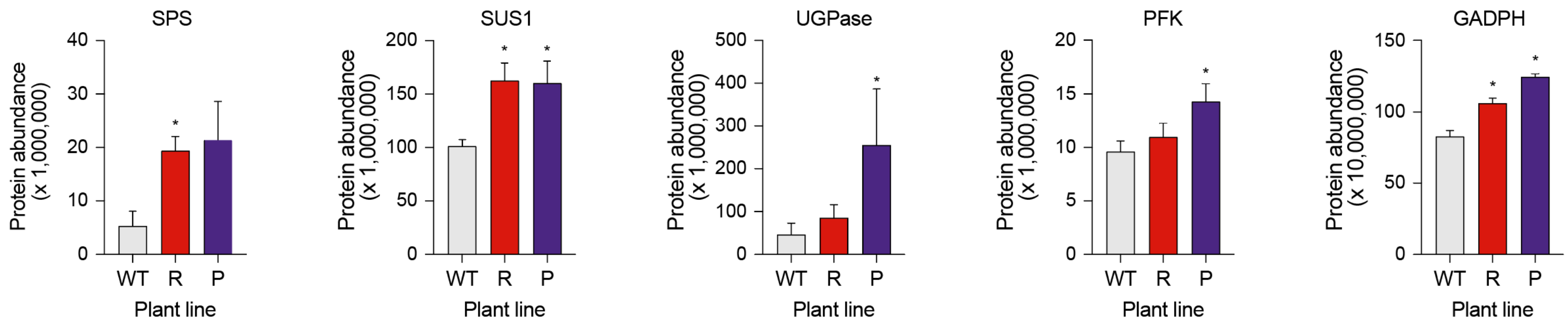

FIGURE S2 Complement to Figure 4–Relative abundance of selected sucrose-processing and glycolytic enzymes upregulated in mutant lines ΔCCD7–R and ΔCCD8–P. Each value is the mean of four biological replicates ± SE. Asterisks (*) indicate statistically significant differences compared to the wild-type line (WT) (post-ANOVA Dunnett's test; *, $p < 0.05$). Non-abbreviated enzyme names and detailed information on protein abundances are provided in supplemental **Table S1**.

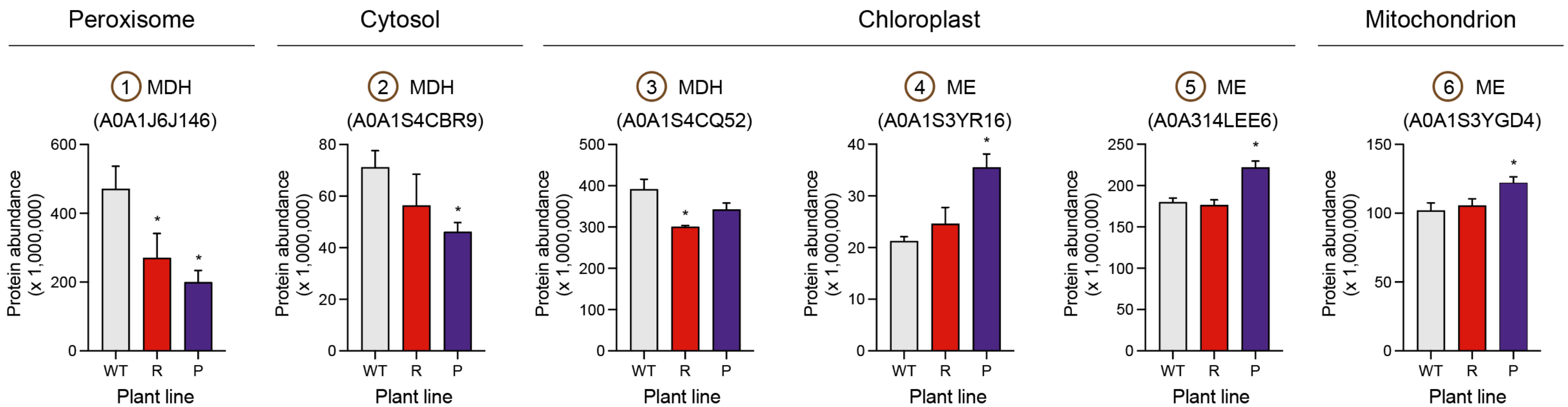

FIGURE S3 Complement to Figure 5–Relative abundance of malate dehydrogenase (MDH) and malic enzyme (ME) isoforms in mutant lines ΔCCD7–R and ΔCCD8–P. Each value is the mean of four biological replicates ± SE. Asterisks (*) indicate statistically significant differences compared to the wild-type line (WT) (post-ANOVA Dunnett's test; *, $p < 0.05$). Details on regulation trends for the MDH and ME isoforms are provided in supplemental **Table S2**.

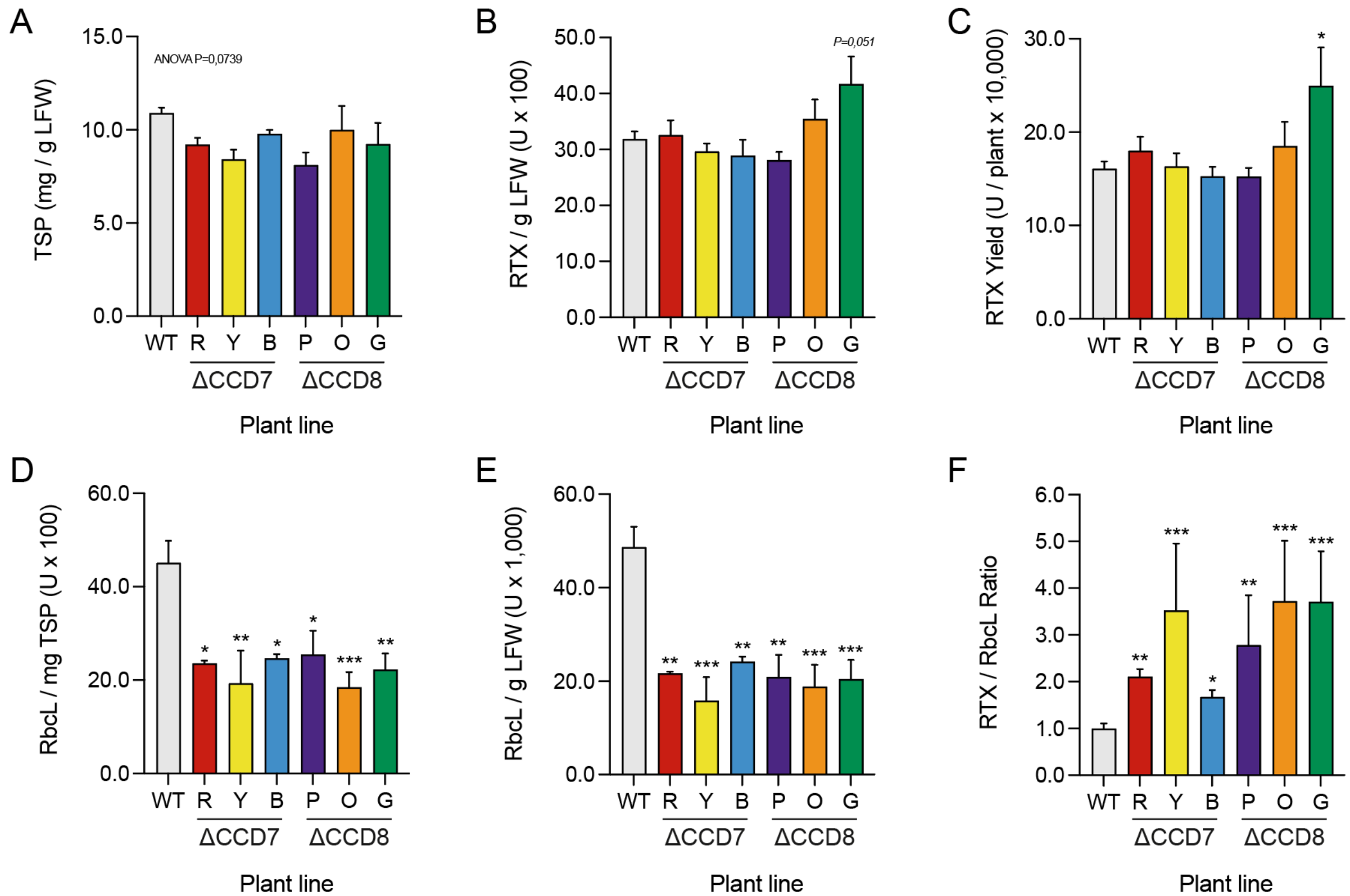

FIGURE S4 Transient expression of chimeric antibody rituximab (RTX) in wild-type line (WT) and mutant lines ΔCCD7–R, ΔCCD7–Y, ΔCCD7–B, ΔCCD8–P, ΔCCD8–O and ΔCCD8–G. **A** Total soluble protein (TSP) content per gram leaf fresh weight at the end of the expression period. **B** Rituximab content per gram leaf fresh weight. **C** Rituximab yield per plant. **D** Rubisco large subunit (RbcL) specific content per mg TSP. **E** RbcL ponderal content per gram leaf fresh weight. **F** Rituximab to RbcL content ratio in agroinfiltrated leaves compared to the WT (arbitrary value of 1.0). All samples were harvested 6 days post-infiltration. Values on this figure are the mean of three or four biological replicates ± SE. Asterisks (*) indicate statistically significant differences compared to the WT (post-ANOVA Dunnett's test; *, $p < 0.05$ / **, $p < 0.01$ / ***, $p < 0.001$).
