# Supplementary material for: Gene Editing of Nicotiana benthamiana Architecture for Space‐Efficient Production of Recombinant Proteins in Closed Environments
Source: Plant Biotechnol J. 2026 May 14:10.1111/pbi.70685. Online ahead of print. doi: 10.1111/pbi.70685 (PMC13399606; doi:10.1111/pbi.70685)
Supplement: Supplementary file 2 — Table S1: Sucrose processing, glycolytic, TCA cycle and oxidative phosphorylation enzymes and proteins up‐ or downregulated in mutant lines ΔCCD7‐R and ΔCCD8‐P. Table S2: Malate‐processing enzymes up‐ or downregulated in mutant lines ΔCCD7–R and ΔCCD8–P. [file PBI-9999-0-s004.pdf]

Table S1 Sucrose processing, glycolytic, TCA cycle and oxidative phosphorylation enzymes and proteins up- or downregulated in mutant lines ΔCCD7-R and ΔCCD8-P. [1]

UNIPROT No. [2]	Protein	Abbreviation	EC No.	Cell location	ΔCCD7-R [3]	ΔCCD8-P
	**Sucrose processing**					
A0A1J6I8F8	Sucrose phosphate synthase	SPS	2.4.1.14	Cytosol	**1.873**	2.015
A0A1J6IAM7	Sucrose synthase (SUS1)	SUS1	2.4.1.13	Cytosol	**0.682**	**0.662**
A0A1S3YUU6	UDP-glucose pyrophosphorylase	UGPase	2.7.7.9	Cytosol	0.880	**2.458**
A0A314LE77	Phosphoglucomutase	PGM	5.4.2.2	Cytosol	0.128	**0.458**
F2Z9R3	Glucose-6-P dehydrogenase	G6PDH	1.1.1.49	Cytosol	**0.426**	**0.554**
A0A1J6L3E7	Invertase (β-fructofuranosidase)	INV	3.2.1.26	Vacuole	–	**0.321**
	**Glycolysis – Phase 1**					
Q9SEK2	Hexokinase-1	HXK1	2.7.1.1	Cytosol	-0.312	**0.645**
A0A1S4CI25	Phosphohexose isomerase	GPI	5.3.1.9	Cytosol	0.180	**0.415**
A0A1S3YDQ2	6-phosphofructokinase (ATP-dependent)	PFK	2.7.1.11	Cytosol	0.192	**0.574**
A0A1J6IGW4	Fructose-biphosphate aldolase (Aldolase)	FBA	4.1.2.13	Cytosol	0.244	**0.571**
	**Glycolysis – Phase 2**					
B3F8I0	Glyceraldehyde-3-P dehydrogenase	GAPDH	1.2.1.12	Cytosol	**0.357**	**0.589**
A0A1S4BTZ7	Phosphoglycerate kinase	PGK	2.7.2.3	Cytosol	0.204	**0.472**
A0A1S3XWD3	Phosphoglycerate mutase	iPGAM	5.4.2.12	Cytosol	0.151	**0.282**
A0A1J6ITL2	Pyruvate kinase	PK	2.7.1.40	Cytosol	–	**0.335**

Table S1 Cont'd.[1]

UNIPROT No. [2]	Protein	Abbreviation	EC No.	Cell location	ΔCCD7-R [3]	ΔCCD8-P
	**Tricarboxylic cycle**					
	PYRUVATE DEHYDROGENASE COMPLEX	PDC				
A0A1S4B2J2	*Pyruvate dehydrogenase E1 component, subunit β*	PDHB	1.2.4.1	Mitochondrion	**0.214**	**0.363**
A0A1S4AK33	*Pyruvate dehydrogenase E1 component, subunit α*	PDHA	1.2.4.1	Mitochondrion	0.111	**0.222**
A0A1S4C175	*Dihydrolipoamide acetyltransferase E2 component*	E2	2.3.1.12	Mitochondrion	**0.498**	0.572
A0A1S3Y542					**0.273**	**0.364**
					**0.260**	**0.343**
					**0.218**	0.148
A0A1S3WZQ8	Aconitase hydratase (aconitase)	ACO	4.2.1.3	Mitochondrion	**0.259**	**0.469**
A0A314KP37					**0.208**	**0.371**
A0A1U7W1S4	Isocitrate dehydrogenase	IDH	1.1.1.42	Mitochondrion	–	**0.248**
A0A1J6IKJ3					0.156	**0.248**
A0A1U7WU40					0.148	**0.230**
A0A1S3YKI8	Succinate dehydrogenase [4]	SDH	1.3.5.1	Mitochondrion	0.082	**0.424**
A0A1J6J5W3					0.080	**0.416**
A0A1J6IUY6	Malate dehydrogenase	MDH	1.1.1.37	Mitochondrion	**-0.290**	0.097
	**Oxidative phosphorylation**					
	**Complex I** – NADH DEHYDROGENASE	NDH	7.1.1.2	Mitochondrion		
A0A1S4AAX1	*Flavoprotein 1*	NDUFV1			**0.448**	**0.727**
A0A1S4BIG4	*Iron-sulfur protein 1*	NDUFS1			**0.285**	**0.513**
Q5M9T3	*Iron-sulfur protein 3*	NDUFS3			0.075	**0.441**

Table S1 Cont'd.[1]

UNIPROT No. [2]	Protein	Abbreviation	EC No.	Cell location	ΔCCD7-R	ΔCCD8-P
	**Oxidative phosphorylation (Cont'd)**					
	**Complex II** – SUCCINATE DEHYDROGENASE [4]	SDH	1.3.5.1	Mitochondrion		
A0A1S3YKI8	*Flavoprotein subunit*	SDHA			0.082	**0.424**
A0A1J6J5W3	*Iron-sulfur subunit*	SDHB			0.080	**0.416**
A0A1J6J759	**Complex III** – Cytochrome bc1 complex, sub. 7	QCR7 (UQCRB)	N/A	Mitochondrion	0.172	**0.358**
A0A314KZN8	Cytochrome c	Cyt c	N/A	Mitochondrion	**0.431**	**0.617**
A0A1S3WZA4	**Complex V** – ATP synthase subunit O	OSCP	N/A	Mitochondrion	–	**0.339**

[1] Data in the last two columns are expressed as log2 ratio values for either mutant compared to the control strain. Positive values indicate an upregulation in the mutant line(s), negative values a downregulation. All enzymes in the table were detected in the two ΔCCD lines and significantly regulated (**in bold**) in at least one line (limma p-values < 0.05).

[2] UniProt accession numbers retrieved from the UniProt consortium database (https://www.uniprot.org), Dec. 2024 to Mar. 2025.

[3] Absolute log2 values smaller than 0.070 (<|5%| change) were not included in the table (– sign).

[4] Enzyme located in the mitochondrial inner membrane; participates in both oxidative phosphorylation (Complex II) and the tricarboxylic cycle.

Table S2 Malate-processing enzymes up- or downregulated in mutant lines ΔCCD7–R and ΔCCD8–P.[1]

UNIPROT No. [2]	EC Number	Cellular location [3]	ΔCCD7-R [4]	ΔCCD8-P
**MALATE DEHYDROGENASE**				
A0A1J6J146	1.1.1.37	Peroxisome	**-0.801**	**-1.235**
A0A1S4CBR9		Cytosol	-0.337	**-0.625**
A0A1S4CQ52		Chloroplast	**-0.380**	-0.194
A0A1J6IUY6		Mitochondrion	**-0.290**	0.097
**MALIC ENZYME**				
A0A1S3YR16	1.1.1.40	Chloroplast	0.212	**0.742**
A0A1S3Y729			–	**0.292**
A0A314LEE6			–	**0.305**
A0A1S4B7M0	1.1.1.39	Mitochondrion	–	**0.242**
A0A1S3YGD4			–	**0.258**
A0A1J6I8D1			–	**0.250**

[1] Data in the last two columns are expressed as log2 ratio values for either mutant compared to the control strain. Positive values indicate an upregulation in the mutant line(s), negative values a downregulation. All enzyme isoforms in the table were detected in the two ΔCCD lines and significantly regulated (**in bold**) in at least one line (limma p-values < 0.05).

[2] UniProt accession numbers retrieved from the UniProt consortium database (https://www.uniprot.org), Dec. 2024 to Mar. 2025.

[3] As determined from available information in Uniprot for each isoform or related isoforms in Arabidopsis, and reconfirmed by sequence analysis inferences using the subcellular prediction program Deep Loc 2.0 (https://services.healthtech.dtu.dk/services/DeepLoc-2.0/).

[4] Absolute log2 values smaller than 0.070 (<|5%| change) were not included the table (– sign).
